# Myristyltrimethylammonium Bromide (MYTAB) as a Cationic Surface Agent to Inhibit *Streptococcus mutans* Grown over Dental Resins: An In Vitro Study

**DOI:** 10.3390/jfb11010009

**Published:** 2020-02-15

**Authors:** Paola Andrea Mena Silva, Isadora Martini Garcia, Julia Nunes, Fernanda Visioli, Vicente Castelo Branco Leitune, Mary Anne Melo, Fabrício Mezzomo Collares

**Affiliations:** 1 Dental Materials Laboratory, School of Dentistry, Federal University of Rio Grande do Sul, 90035-003 Porto Alegre-RS, Brazil; pao_mena100@hotmail.com (P.A.M.S.); isadora.garcia@ufrgs.br (I.M.G.); vicente.leitune@ufrgs.br (V.C.B.L.); 2 Universidad Central del Ecuador, 170129 Quito, Ecuador; 3 Postgraduate Department, Universidad Regional Autónoma de Los Andes, 170129 Quito, Ecuador; 4 Oral Pathology Department, School of Dentistry, Federal University of Rio Grande do Sul, 90035-003 Porto Alegre-RS, Brazil; jusnunes@icloud.com (J.N.); fernanda.visioli@ufrgs.br (F.V.); 5 Operative Dentistry Division, General Dentistry Department, University of Maryland School of Dentistry, Baltimore, MD 21201, USA; 6 Ph.D. Program in Biomedical Sciences, University of Maryland School of Dentistry, Baltimore, MD 21201, USA

**Keywords:** dental materials, dentistry, anti-bacterial agents, dental caries, biocompatible materials, biofilms, quaternary ammonium compounds

## Abstract

This in vitro study evaluated the effect of myristyltrimethylammonium bromide (MYTAB) on the physical, chemical, and biological properties of an experimental dental resin. The resin was formulated with dental dimetacrylate monomers and a photoinitiator/co-initiator system. MYTAB was added at 0.5 (G_0.5%_), 1 (G_1%_), and 2 (G_2%_) wt %, and one group remained without MYTAB and was used as the control (G_Ctrl_). The resins were analyzed for the polymerization kinetics, degree of conversion, ultimate tensile strength (UTS), antibacterial activity against *Streptococcus mutans*, and cytotoxicity against human keratinocytes. Changes in the polymerization kinetics profiling were observed, and the degree of conversion ranged from 57.36% (±2.50%) for G_2%_ to 61.88% (±1.91%) for G_0.5%_, without a statistically significant difference among groups (*p* > 0.05). The UTS values ranged from 32.85 (±6.08) MPa for G_0.5%_ to 35.12 (±5.74) MPa for G_Ctrl_ (*p* > 0.05). MYTAB groups showed antibacterial activity against biofilm formation from 0.5 wt % (*p* < 0.05) and against planktonic bacteria from 1 wt % (*p* < 0.05). The higher the MYTAB concentration, the higher the cytotoxic effect, without differences between G_Ctrl_ e G_0.5%_ (*p* > 0.05). In conclusion, the addition of 0.5 wt % of MYTAB did not alter the physical and chemical properties of the dental resin and provided antibacterial activity without cytotoxic effect.

## 1. Introduction

Dental caries-linked bacteria growing in biofilms play a pivotal role in the initial formation and development of carious lesions. The demand for the development of antibacterial surfaces has gained prominence in order to reduce patients’ susceptibility to new or repeated diseases [1,2,3,4,5]. In dentistry, dental materials have been developed with antibacterial agents to provide new preventive and treatment dynamics for patients [6,7,8,9]. The antibacterial approach for dental materials relies on biological interactions of the bacteria grown over the materials, and the contact with antibacterial agents presents in the surface or is released by the materials [10,11,12,13]. In this context, the achievement of surfaces able to reduce caries-linked biofilm formation over dental tissues or restorative structures has gained the attention of dental biomaterial researchers.

The current understanding of the dental caries disease process and the new advances in dental materials promote the preservation of dental structure and the application of minimally invasive techniques [14]. Under this framework, dental resins are the first option for the replacement of dental hard tissues that were lost due to carious lesions [15,16]. Dental resins are materials cured by chemical or physicochemical (via photo-activation) processes, and they present inorganic fillers depending on their purpose. In areas where it is necessary to increase strength (occlusal/chewing surfaces of teeth), a high amount of inorganic fillers is incorporated [17]. Dental resins with low viscosity can also be successfully used to seal the biting surfaces of teeth, decreasing the incidence of caries lesions [18].

The oral environment provides many challenges to the physical and chemical stability of dental resins, such as high humidity, temperature, and pH variations [19,20]. Likewise, acids leached by high acidogenic caries-linked bacteria such as *Streptococcus mutans*, conjointly with a degradative attack of enzymes, inherently present in the saliva, can jeopardize the materials’ properties over time [21]. More expressively, acidic attack from high acidogenic caries-linked bacteria is a crucial step in tooth demineralization around the restorations [22]. On this basis, the search for long-lasting strategies challenging biofilm accumulation on dental resins, as well as the consequent caries lesion development around restorations, must be addressed.

Antibacterial surfaces with a bacteria-killing function have shown great promise in biological and biomedical applications, in particular for dental resin-based materials. This approach employs the incorporation of quaternary ammonium compounds (QACs) in the monomeric blend of the resin-based material formulation [9,11,23]. QACs have facile synthesis, antibacterial property, and a lack of a detrimental effect on the mechanical and physical properties when incorporated in concentrations. Typically, QAS exhibits a positive charge, which confers the surface with the ability to attach and kill bacteria efficiently. The killing effect is attributed to their electronically interaction and linking to bacteria membrane and wall, along with their possible diffusion into the cytoplasmic membrane, an increase of osmotic pressure, and the release of some cytoplasmic constituents [24,25].

Myristyltrimethylammonium bromide (MYTAB) is a QAC with an alkyl-chain length of 14 carbons presenting a chemical structure of C_17_H_38_NBr_,_ a molecular weight of 336.39, and a cationic polar head group. Trimethyl alkylammonium compounds such as the MYTAB series can also strongly affect bacterial membrane properties of a wide range of microorganisms, including changes in electrokinetic potential as well as net surface charge [26]. A recent study has shown promising application for the sealing of dental roots during endodontic treatment with expressive bacterial reduction of the endodontic pathogen [27]. Additionally, the long-chain MYTAB presents the potential to act as a useful endocytosis inhibitor for cell biology, inhibiting different forms of endocytosis in multiple cell systems [26].

The present study aimed to evaluate the potential ability of MYTAB to impair bacterial reduction when incorporated into dental resin at increasing concentrations. Its antibacterial effects against *S. mutans*, a pivotal cariogenic pathogen presented in planktonic and biofilm stages, were assessed. The cytotoxicity effect on human keratinocytes was investigated. The chemical and physical effects of its incorporation on the materials were also explored.

## 2. Results

**Figure 1:**
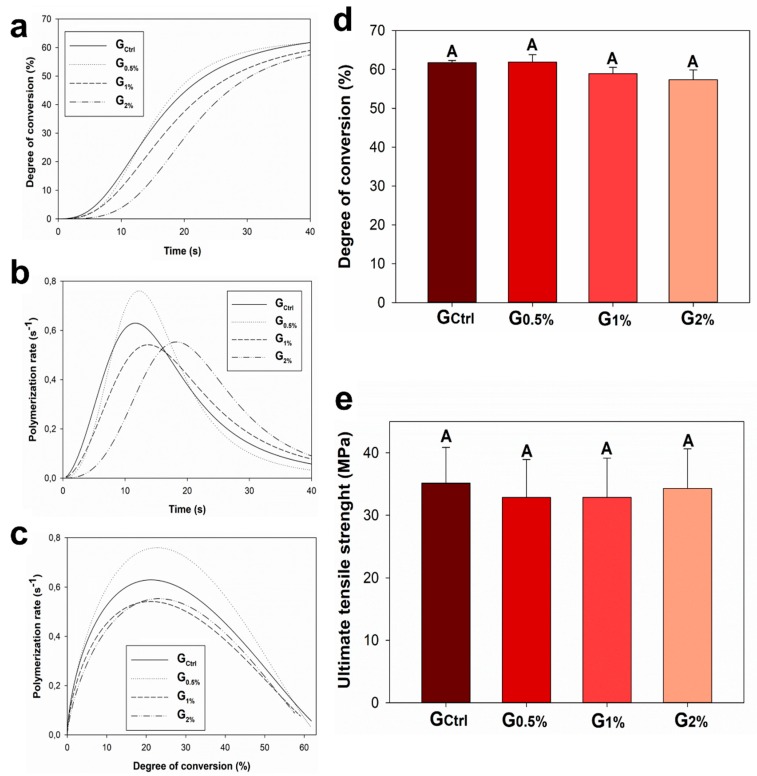
Comprehensive results of polymerization kinetics (**a**–**c**), degree of conversion after 40 s of photoactivation (**d**), and ultimate tensile strength (UTS) (**e**). Same capital letters indicate no statistical difference among groups (*p* > 0.05). The groups had different polymerization kinetics without statistical difference for the degree of conversion and UTS.

**Figure 2:**
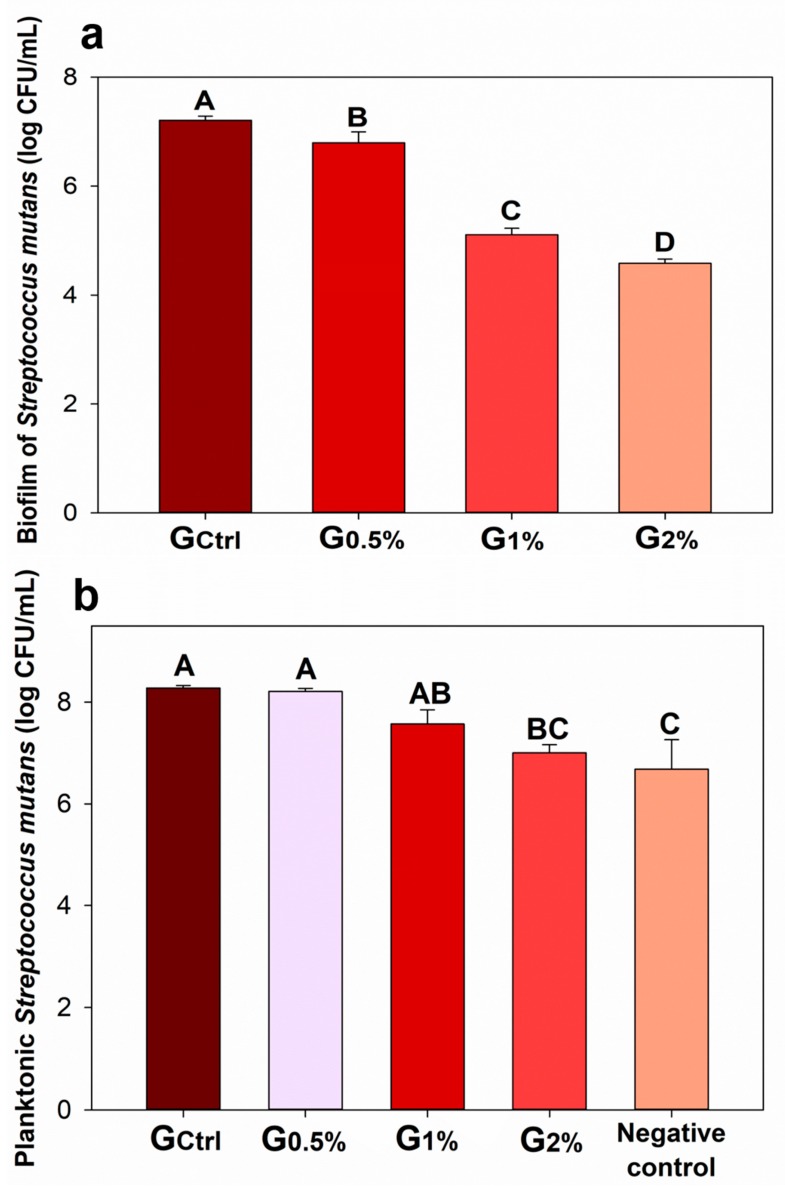
Bacterial colony forming unit counting: (**a**) *Streptococcus mutans* biofilms and (**b**) planktonic *S. mutans* that were in contact with the polymerized samples. Values indicated by different letters indicate statistical differences among the groups (*p* < 0.05).

Figure 1 shows the results of polymerization kinetics (Figure 1a–c), degree of conversion (Figure 1d), and ultimate tensile strength (UTS) (Figure 1e). The experimental dental resins had different polymerization behavior throughout the 40 s of photoactivation. The degree of conversion per time is shown in Figure 1a. The results (Figure 2d) ranged from 57.36% (±2.50%) for G_2%_ to 61.88% (±1.91%) for G_0.5%_, without statistical difference among groups (*p* > 0.05). Figure 1b indicates the results of the polymerization rate per time, showing that the higher the concentration of MYTAB, the higher the delay in achieving the maximum polymerization rate. Moreover, the higher the concentration of MYTAB, the lower the maximum polymerization rate. Figure 1c displays these differences, showing that at the same degree of conversion among groups, the polymerization rate of G_Ctrl_ was higher than G_1%_ and G_2%_ and lower than G_0.5%_. In Figure 1d, the degree of conversion revealed similar behavior for the tested groups (*p* > 0.05). The mechanical property of the dental resins was evaluated under UTS and expressed in MPa, as shown in Figure 1e. The UTS ranged from 32.85 (±6.08) MPa for G_0.5%_ to 35.12 (±5.74) MPa for G_Ctrl_. There was no statistical difference among groups (*p* > 0.05).

Figure 2 shows the results of the antibacterial activity of the experimental dental resins against biofilm formation of *S. mutans* and planktonic *S. mutans*, expressed by the log reduction in colony-forming unit per milliliter. In the microbiological assessment against biofilm formation (Figure 2a), the values ranged from 4.58 (±0.08) log CFU/mL for G_2%_ to 7.21 (±0.08) log CFU/mL for G_Ctrl_ (*p* < 0.05). A greater bacterial reduction (*p* < 0.05) was observed at the higher concentration of MYTAB incorporated into the dental resin. In the test against planktonic bacteria (Figure 2b), the values ranged from 6.68 (±0.58) log CFU/mL for G_2%_ to 8.28 (±0.05) log CFU/mL for G_Ctrl_ (*p* < 0.05). The group presenting MYTAB concentration at 2% expressed the highest *S. mutans* bacterial reduction (*p* < 0.05).

**Figure 3:**
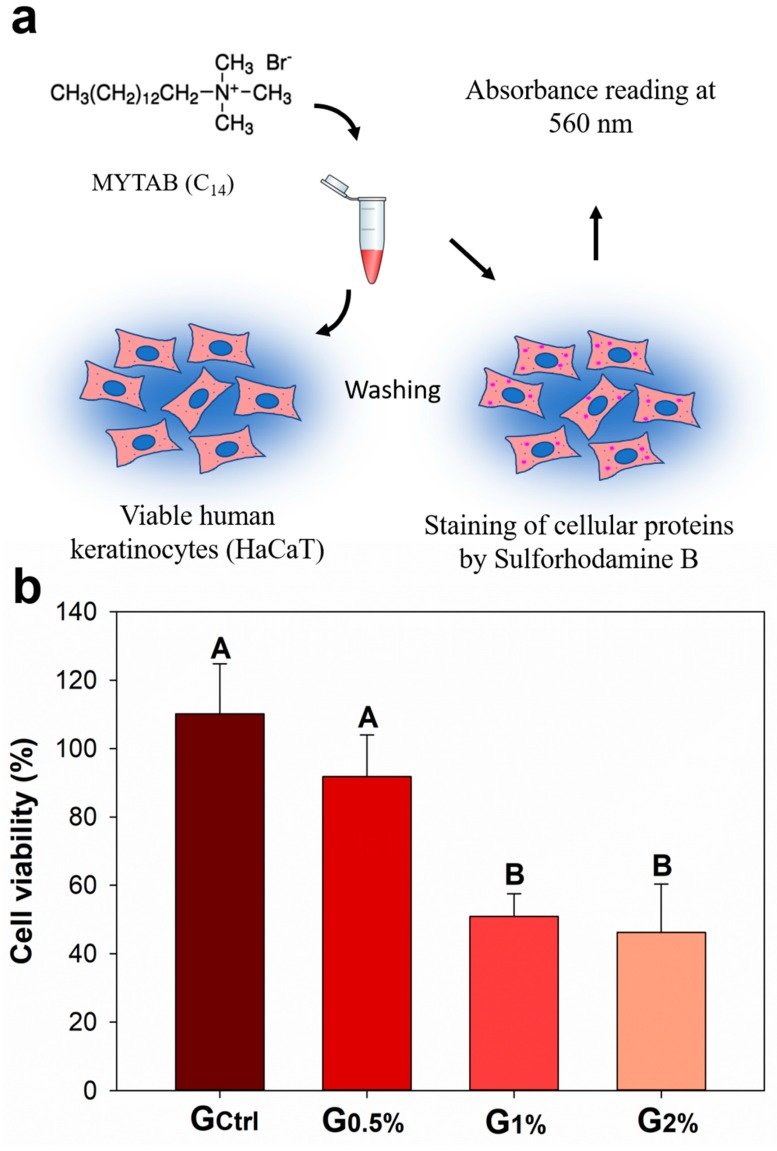
Cytotoxicity evaluation of the experimental dental resins expressed by percentage of cell viability: (**a**) structure of myristyltrimethylammonium bromide (MYTAB) and schematic drawing of sulforhodamine B (SRB) assay; and (**b**) MYTAB cytotoxicity assessed in normal human keratinocytes (HaCaT) line. Different capital letters indicate statistical differences among groups (*p* < 0.05).

Figure 3 presents the effects of MYTAB incorporated into a dental resin on normal human keratinocytes (HaCaT) for cytotoxicity assessed by sulforhodamine B (SRB) assay. The percentage of viability of the cells ranged from 45.26% (±14.11%) for G_2%_ to 110.16% (±14.64%) for G_Ctrl_ (*p* < 0.05). G_0.5%_ (91.82% ± 12.17%) presented no statistical difference in comparison to G_Ctrl_ (*p* > 0.05) for cytotoxicity against human keratinocytes.

## 3. Discussion

Dental resins are reliable materials to restore teeth, and, when used as pit and fissure sealants, could prevent new caries lesions [18,28,29]. Nevertheless, the formation of caries lesions around dental resins is still a major concern due to biofilm accumulation [22,30]. In order to prevent this issue, we investigated the effect of a cationic organic compound, MYTAB, in the properties of an experimental dental resin. The formulated resin is a suitable material for dental restorative purposes.

The longevity of dental restorative materials is strongly related to material rates of polymerization. High monomer conversion is essential for polymers to achieve reliable properties and stability [31]. The study of their polymerization behavior of modified formulations assists in understanding the effects of the incorporation of the compound on the functional aspect of the restorative material. Here, the formulated experimental dental resins showed different polymerization kinetics depending on the concentration of MYTAB. The effects were more evident with the addition of 1 wt % of MYTAB in the resin (Figure 2 A–C). From this concentration, the polymerization process was delayed, and the groups reached the maximum polymerization rate later compared to G_Ctrl_ and G_0.5%_.

On the other hand, G_0.5%_ showed a higher maximum polymerization rate compared to all groups (Figure 2 B). The rationale for this may be attributed to different viscosities among groups [32]. It was previously suggested that cationic surfactants could increase monomer chain mobility and modify polymerization behavior [33]. The lower viscosity and the higher monomer chain mobility for G_0.5%_ may lead to a higher maximum polymerization rate. Nevertheless, by increasing MYTAB incorporation, the spaces among monomer chains would be increased further, leading to a lower polymerization rate for G_1%_ and G_2%_. Despite these events, there was no difference in the degree of conversion among groups, and they achieved high values of conversion, similar to commercial dental resins [34].

Even though all groups presented a reliable degree of conversion, the delay during the polymerization kinetics observed for G_1%_ and G_2%_ could induce the formation of a more linear polymer, with lower crosslinking density [35]. Therefore, the mechanical evaluation of the formulated materials was essential in better understanding their performance. The specimens of dental resins were submitted to tensile strength until fracture in a universal testing machine with no statistical difference among groups. The UTS was a promising outcome because the incorporation of antibacterial agents could lead to lower mechanical properties [36]. The dental resins formulated in this study may be used in occlusal surfaces, where repetitive chewing stress is applied. The maintenance of resins’ mechanical properties besides the antibacterial activity presented is essential in keeping the material in function.

In chewing sites, the experimental material proposed could be used not only for the prevention of caries lesions [18] but also to treat teeth already affected by the disease [29]. Pits and fissures are sites of difficult hygiene, favoring biofilm accumulation. Dental sealants successfully inhibit dental caries due to their ability to seal the demineralized tissue and form a mechanical barrier, inhibiting bacterial growth, hampering lesion progression, or preventing the demineralization of the sealed area [18,37]. The use of dental sealants in children and adolescents decreases the susceptibility to caries development in occlusal surfaces of permanent molars in comparison to people with no sealed teeth. Even among the people with sealed teeth, there are around 18% who present new lesions over time [18]. The composition of the sealants available in the market does not present antibacterial agents in their composition, which could be a strategy to reduce this percentage.

The parental composite resin formulated had its viscosity manually accessed for friendly use in clinical settings as a sealant. In the previous study with similar QAC, a high antibacterial effect of myristyltrimethylammonium bromide against *Enterococcus faecalis* was observed [27]. In both cases, these QACs have a long alkyl chain, which increases the QAC’s hydrophobicity [38]. Consequently, when leached, these agents are more prone to penetrate bacterial walls and membranes [39]. With MYTAB, the higher its concentration in the dental resin, the lower the viability of *S. mutans* in biofilm and in planktonic stages.

For the assessment of antibacterial property, discs of the polymerized dental resins entered into contact with an enriched broth containing *S. mutans* as previously performed [13,33,40,41,42]. This bacterium is Gram-positive, and it is present in intraoral multispecies biofilms [43]. *S. mutans* group is the main bacteria associated with caries lesion development [44], and they can attach to dental and material surfaces [1,21]. The samples were exposed to planktonic as well as biofilm stages. During the contact with a dental resin containing MYTAB, planktonic bacteria reduction is suggested because of the leaching of some MYTAB molecules for the broth. The lack of assessment of the long-term antibacterial effect of the dental resins is a limitation of this study. Besides this, the quantities of MYTAB released over time from the polymerized resin is not known. The knowledge about this event could be valuable in order to understand the long-term behavior of the material and to assist in predicting the in vivo outcomes. However, the main goal of this study was to investigate the potential adverse effects of the addition of this compound into the composite to impart an antibacterial effect.

The cytotoxicity test was performed against human keratinocytes via the SRB method. Through this method, proteins of viable cells are stained, indicating increased viability when higher optical density is achieved [45]. The higher the incorporation of MYTAB, the higher the cytotoxic effect observed. This result corroborates with previous studies, which showed that cationic compounds with long-alkyl chains lead to high cytotoxic effects [46]. The International Organization for Standardization (ISO) recommends that biomaterials must promote up to 70% of cells’ viability in order to not be considered cytotoxic [47]. It is worth mentioning that, despite the lower values of viability found for 1 and 2 wt % of MYTAB, all groups were directly treated with the eluates from the samples. In other words, we did not dilute the agent as other studies do [46], but we did somewhat increase the challenge by using the eluates on human cells during 72 h of contact.

A similar compound to MYTAB, a quaternary ammounium compound called ATAB, has been found to show antibacterial activity against *Enterococcus faecalis*, a relevant bacterium to endodontic infections [48]. The main difference between MYTAB and ATAB is the presence of mixing of QACs with different aliphatic chain lengths for ATAB, which does not occur in the case of MYTAB. In the previous study, ATAB was associated with halloysite nanotubes (HNT) that were incorporated in the sealer without the evaluation of ATAB alone [27], which might have repercussions for physical, chemical, or biological properties. In the same study, there were no cytotoxic effects for pulp cells with the incorporation of 10 wt % of ATAB/ halloysite nanotubes in the sealer, even when the proportion of ATAB/ halloysite nanotubes was 2:1 [27]. Here, MYTAB was not carried out by another system because we aimed to evaluate the QAC itself as a free drug, which may have increased the biological effects. G_0.5%_ showed no cytotoxicity in comparison to G_Ctrl,_ and both promoted viability higher than 70%. Therefore, the addition of 0.5 wt % of MYTAB may be a promising method for providing antibacterial activity for a dental resin without compromising its physical, chemical, and biological properties. The material here formulated is an exciting approach to be further translated for clinical trials.

## 4. Materials and Methods

### 4.1. Study Design and Formulation of Dental Resins

**Figure 4:**
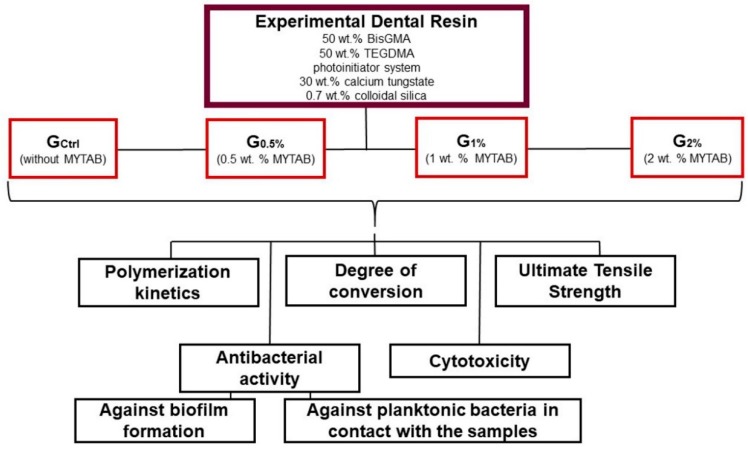
Flowchart of the study design. Experimental dental resins were formulated with different concentrations of a quaternary ammonium compound (MYTAB). Bisphenol A glycol dimethacrylates (BisGMA) and triethylene glycol dimethacrylate (TEGDMA) were used for the monomeric blend. The four experimental groups were evaluated for chemical, physical, and antibacterial properties, alongside their effect against human keratinocytes.

The study design is described in the flowchart presented in Figure 4. All reagents of the analytical grade for in vitro experiments were purchased from Sigma-Aldrich (Sigma-Aldrich Chemical Company, St. Louis, MO, USA) if not otherwise specified. First, a parental resin was formulated with two dimethacrylate monomers: bisphenol A glycol dimethacrylates (BisGMA) and triethylene glycol dimethacrylate (TEGDMA), at the proportion of 1:1. As a photoinitiator/co-initiator system, camphorquinone and ethyl 4-dimethylaminobenzoate were added to the resin at 1 mol % each. Butylated hydroxytoluene was added at 0.01 wt % as a polymerization inhibitor. Calcium tungstate (CaWO_4_) was added as inorganic filler at 30 wt %. Colloidal silicon dioxide (SiO_2_; Aerosil 200, Evonik, Essen, Germany) was incorporated at 0.7 wt % to adjust the resin’s viscosity. After being hand-mixed for 5 min, they were sonicated for 180 s and hand-mixed for 5 min.

MYTAB with a purity of > 99% was added to the parental dental resin formulation at an increased double concentration of 0.5, 1, and 2% wt% mass fractions.

### 4.2. Polymerization Kinetics and Degree of Conversion

Fourier transform infrared spectroscopy (FTIR, Vertex 70, Bruker Optics, Ettinger, Germany) was used to evaluate the polymerization kinetics and the degree of conversion of the experimental dental resin. For this test, three samples per group were analyzed by dispensing them on the attenuated total reflectance (ATR) device in the polyvinylsiloxane matrix measuring 1 mm thickness and 4 mm in diameter. To perform the photoactivation of each sample, the light-cured unit (Radii Cal, SDI, Australia; 1200 mW/cm^2^) was fixed using support to maintain 1 mm between the tip of the light-cured unit and the top of each sample. During the 40 s of photoactivation, two spectra were obtained per second in absorbance mode (10 kHz velocity, 4 cm^−1^ resolution; Opus 6.5 software, Bruker Optics, Ettlingen, Germany) in the range of 4000 to 400 cm^−1^. The first spectrum obtained was used as “uncured resin dental resin”, and the last spectrum as “cured resin dental resin” in the calculation of the degree of conversion. The peak at 1610 cm^−1^ from aromatic carbon–carbon double bond was used as an internal standard, and the peak at 1640 cm^−1^ was used as an aliphatic carbon–carbon double bond to calculate the conversion in percentage (Equation (1)) [31]. The polymerization rate was calculated using the degree of conversion at time *t* subtracted from the degree of conversion at time *t-1*.

Equation (1):

(1) $$\begin{array}{l} DC\;\left(\%\right)\\ =100\times \left(\frac{\mathrm{peak}\;\mathrm{height}\;\mathrm{of}\;\mathrm{cured}\;\mathrm{aliphatic}\;C=C/\mathrm{peak}\;\mathrm{height}\;\mathrm{of}\;\mathrm{cured}\;\mathrm{aromatic}\;C=C}{\mathrm{peak}\;\mathrm{height}\;\mathrm{of}\;\mathrm{uncured}\;\mathrm{aliphatic}\;C=C/\mathrm{peak}\;\mathrm{height}\;\mathrm{of}\;\mathrm{uncured}\;\mathrm{aromatic}\;C=C}\right) \end{array}$$

### 4.3. Ultimate Tensile Strength (UTS)

Ten samples per group with hourglass shape were prepared in a metallic matrix that was 8.0 mm long, 2.0 mm wide, and of 1.0 mm thickness, with a cross-sectional area of ±1 mm^2^. Each uncured sample was placed in the mold and photoactivated for 20 s on each side (bottom and top). The prepared samples were stored in distilled water at 37 °C for 24 h. The samples were fixed in jigs with cyanoacrylate resin, and they were submitted under tensile strength in a universal testing machine (EZ-SX Series, Shimadzu, Kyoto, Japan) at 1 mm/min until fracture. The maximum force value (Newtons, N) achieved was divided by the constriction area of each sample, which was measured with a digital caliper (Mitutoyo, Kawasaki, Kanagawa, Japan) to calculate the maximum value of tensile strength (Equation (2)). The results were expressed in megapascals (MPa).

Equation (2):

(2) $$UTS\;\left(MPa\right)=\left(\frac{Force\;\left(N\right)}{Constriction\;area\;(mm^{2})}\right)$$

### 4.4. Antibacterial Activity

Two in vitro assessments were performed to evaluate the antibacterial activity of the experimental dental resin: (1) planktonic bacteria and (2) against biofilm formation on the polymerized samples. The bacteria used in both tests were *S. mutans* (NCTC 10449). Three samples per group were prepared for the biofilm test, and the other three samples per group were used in the test against planktonic bacteria. *S. mutans* were prepared according to a previous study [13], and the initial inoculum used for the tests was assessed by serial dilution method and colony counting, which indicated an inoculum at 7.8 × 10^7^ CFU/mL.

To evaluate the antibacterial activity against biofilm formation, the polymerized samples (1 mm thickness and 4 mm diameter) were fixed on Teflon specimens that were fixed on the cover of a 48-well plate, and this assembly was sterilized with hydrogen peroxide plasma (58%, 48 min, 56 °C) [13,49]. From the initial inoculum, 100 μL was added in each well of a 48-well plate with 900 μL of brain–heart infusion (BHI) broth with 1 wt % of sucrose. All reagents used in the antibacterial activity analysis were purchased from Aldrich Chemical Company (St. Louis, MO, USA). The sterile set of cover and samples was joined with this 48-well plate and kept for 24 h under 37 °C for biofilm formation on the samples. After this period, each sample was detached from the cover and vortexed for 1 min in 1 mL of sterile saline solution. The solution was serial diluted up to 10^−6^ mL and plated on Petri dishes containing BHI agar to count the colonies and to calculate (Equation 3) the colony-forming units per milliliter (CFU/mL).

Equation (3):

(3) $$CFU/mL=\left(\frac{\mathrm{Avarage}\;\mathrm{number}\;\mathrm{of}\;\mathrm{colonies}\;x\;\mathrm{Dilution}\;\mathrm{factor}}{\mathrm{Volume}\;\mathrm{of}\;\mathrm{culture}\;\mathrm{plate}}\right)$$

To evaluate the antibacterial activity against planktonic bacteria, the BHI broth that was in contact with the polymerized samples along the 24 h mentioned above was used. From each well of the 48-well plate, 100 µL was collected after the 24 h of bacteria–sample contact and was inserted into Eppendorf tubes with 900 µL of saline solution to be vortexed, diluted until 10^−6^, and plated on BHI agar Petri dishes. For this test, an additional group was added as a negative control. The negative control was composed of BHI broth and *S. mutans* at the same proportion (10% of initial inoculum of bacteria in each well) without samples’ contact. Colonies were visually counted, and the results were also expressed in CFU/mL.

### 4.5. Cytotoxicity

To evaluate the possible cytotoxic effect of the experimental dental resin, human keratinocytes (HaCaT, CLS Cell Lines Service GmbH, Eppelheim, Germany) were used in this test [40]. All reagents used to evaluate the possible cytotoxicity of the dental resins were purchased from Aldrich Chemical Company (St. Louis, MO, USA). The keratinocytes were kept in contact (5 × 10^3^ cells/per well) with 100 µL of Dulbecco’s modified Eagle’s medium (DMEM) in 96-well plates for 24 h at 37 °C. On the same day, five samples per group (1 mm thickness and 4 mm diameter) were placed separately in Eppendorf tubes containing 1 mL of DMEM and kept for 24 h at 37 °C. Thus, eluates from possible leaching from the samples were formed. These eluates (100 µL) were kept in contact with the keratinocytes in the 96-well plates for 72 h at 37 °C. There was one group that was maintained without eluates with 100 µL of pure DMEM that was used as a control for the test. In addition to using five samples per group, the eluates were applied in five replications, totalizing 100 wells containing eluates from the samples. After 72 h, 50 μL of trichloroacetic acid/distilled water solution (50:50) was added in each well and kept at 4 °C for 1 hour to fix the cells on the bottom. Running water (30 s) was used to wash the 96-well plates six times. The 96-well plates were kept at room temperature until drying.

Sulforhodamine B at 0.4% (50 μL) was added in each well, and the 96-well plates were kept at room temperature for 30 min. The 96-well plates were washed four times with acetic acid at 1% and kept at room temperature until drying. Trizma solution at 10 mM (100 μL) was added in each well, and the 96-well plates were incubated for 1 hour at room temperature. The absorbance of plates’ wells was analyzed at 560 nm, and the cell viability of the wells that contained eluates was compared to the wells without eluates. The viability of the keratinocytes used was normalized against the viability of cells without contact with eluates (negative control). The results were expressed in percentage using the viability of negative control as 100%.

### 4.6. Statistical Analysis

Data normality was evaluated by the Shapiro–Wilk test. One-way ANOVA and Tukey’s post-hoc test was used to compare groups for all tests at a level of 0.05 of significance.

## 5. Conclusions

The present study incorporated a not yet investigated quaternary ammonium MYTAB into a dental resin, which achieved potent antibacterial effects against dental plaque microcosm biofilms for the first time. The results showed that (1) MYTAB at 2% greatly decreased *S. mutans* in planktonic and biofilm stages compared to G_Ctrl_, (2) higher MYTAB mass fraction rendered the adhesive more strongly antibacterial, (3) MYTAB at 1% mass fraction greatly reduced the cell viability, and (4) the incorporation of MYTAB at a maximum concentration of 2% had no detrimental effect on the degree of conversion and ultimate tensile strength. MYTAB-containing dental resin is a promising strategy for dental applications.
